# Novel Synthetic Polyamines Have Potent Antimalarial Activities *in vitro* and *in vivo* by Decreasing Intracellular Spermidine and Spermine Concentrations

**DOI:** 10.3389/fcimb.2019.00009

**Published:** 2019-02-14

**Authors:** Kamal El Bissati, Henry Redel, Li-Min Ting, Joseph D. Lykins, Martin J. McPhillie, Rajendra Upadhya, Patrick M. Woster, Nigel Yarlett, Kami Kim, Louis M. Weiss

**Affiliations:** ^1^Department of Ophthalmology and Visual Science, The University of Chicago, Chicago, IL, United States; ^2^Department of Medicine, Albert Einstein College of Medicine and Montefiore Medical Center, Bronx, NY, United States; ^3^Department of Internal Medicine and Department of Emergency Medicine, Virginia Commonwealth University Health System, Richmond, VA, United States; ^4^School of Chemistry, University of Leeds, Leeds, United Kingdom; ^5^Department of Drug Discovery and Biomedical Sciences, Medical University of South Carolina, Charleston, SC, United States; ^6^Haskins Laboratories, Department of Chemistry and Physical Sciences, Pace University, New York, NY, United States; ^7^Department of Pathology, Albert Einstein College of Medicine and Montefiore Medical Center, Bronx, NY, United States

**Keywords:** polyamine, *Plasmodium*, malaria, spermidine, spermine, spermidine synthase, thiourea

## Abstract

Twenty-two compounds belonging to several classes of polyamine analogs have been examined for their ability to inhibit the growth of the human malaria parasite *Plasmodium falciparum in vitro* and *in vivo*. Four lead compounds from the thiourea sub-series and one compound from the urea-based analogs were found to be potent inhibitors of both chloroquine-resistant (Dd2) and chloroquine-sensitive (3D7) strains of *Plasmodium* with IC_50_ values ranging from 150 to 460 nM. In addition, the compound RHW, *N*1,*N*7-bis (3-(cyclohexylmethylamino) propyl) heptane-1,7-diamine tetrabromide was found to inhibit Dd2 with an IC_50_ of 200 nM. When RHW was administered to *P. yoelii*-infected mice at 35 mg/kg for 4 days, it significantly reduced parasitemia. RHW was also assayed in combination with the ornithine decarboxylase inhibitor difluoromethylornithine, and the two drugs were found not to have synergistic antimalarial activity. Furthermore, these inhibitors led to decreased cellular spermidine and spermine levels in *P. falciparum*, suggesting that they exert their antimalarial activities by inhibition of spermidine synthase.

## Introduction

Malaria is a global health threat, especially in the developing world. *Plasmodium falciparum* causes the most lethal form of the disease. It is responsible for a high number of clinical cases and deaths annually. About 3.2 billion people remain at risk of malaria. In 2015 alone, there was an estimated 214 million new cases of malaria and 438,000 deaths (World Health Organization, 2015). The number of malaria cases fell from an estimated 262 million in 2000 to 214 million in 2015 (World Health Organization, 2015), due to employment of artemisinin and drug-impregnated bed nets (White et al., 2014; World Health Organization, 2015). Artemisinin-based combination therapies ACTs have become widely adopted as first-line treatment in almost all countries where malaria is endemic (White, 2008). However, recent studies report decreases in parasite clearance rates following artesunate monotherapy or artesunate-mefloquine combination therapy in Thailand and Cambodia, suggesting increasing resistance to this therapeutic approach (Dondorp et al., 2009; Amaratunga et al., 2012; Phyo et al., 2012; Ferreira et al., 2013). In light of the challenge posed by resistant strains of *P. falciparum*, development of new drugs to combat this infection is increasingly necessary and will save lives.

The intraerythrocytic life cycle of *P. falciparum* is dynamic, with the parasite undergoing numerous morphological and physiological transformations throughout the course of infection. Whilst actively dividing, the parasite is capable of generating 36 daughter parasites within 2 days. Innumerable parasitic cellular processes offer diverse chemotherapeutic targets for the inhibition of parasite replication and, thereby, abrogation of disease. Among these targets, is the biosynthesis of polyamines. There is a 10 to 20-fold increase in polyamine levels when the parasite transitions from ring stage to schizonts in infected erythrocytes and there is evidence of parasitic dependence on the polyamine pathway for intraerythrocytic development (Assaraf et al., 1984; Gupta et al., 2005). Moreover, inhibitors of this pathway cause decreased polyamine levels, which, in turn, results in transcriptional arrest (van Brummelen et al., 2009). Consequently, the polyamine pathway appears to be a valid target for further exploration and small molecular development, given its essential role in parasite survival. Polyamines, which include putrescine, spermidine, spermine, and cadaverine (an analog of putrescine), are amine-containing, cationic, low molecular mass compounds, and ubiquitous in eukaryotes and prokaryotes. These polycation compounds interact electrostatically with anionic macromolecules like RNA, DNA, ATP, proteins, and phospholipids (Igarashi and Kashiwagi, 2000; Wallace et al., 2003). These interactions regulate replication, transcription, membrane biogenesis, maintenance of chromatin conformation, specific gene expression, ion channels, and confer protection of nucleic acids against oxidative stress (Igarashi and Kashiwagi, 2000; Wallace et al., 2003). Synthesis of polyamines in most cells is initiated by the production of putrescine from ornithine through the activity of ornithine decarboxylase (ODC). The addition of either one or two aminopropyl groups to the terminal amino groups of putrescine form spermidine and spermine, respectively via *S*-adenosylmethionine (AdoMet) decarboxylase. Most animal and yeast cells can take up polyamines and convert them back to spermidine and putrescine via spermidine/spermine-*N*-acetyltransferase and polyamine oxidase (Pegg and McCann, 1982; Marton and Pegg, 1995). *P. falciparum* lacks the capacity for polyamine interconversion, and the parasite controls polyamine levels exclusively through *de novo* spermidine synthase, which determines levels of spermidine (Müller et al., 2001; Clark et al., 2010). This enzyme has the additional unique function of producing low levels of spermine (Haider et al., 2005).

Many inhibitors of both ODC and AdoMetDC have been synthesized, with the goal of interfering with polyamine metabolism in tumor cells as anti-cancer therapy and prevention (Marton and Pegg, 1995; Casero and Marton, 2007). DFMO (alpha-difluoromethylornithine) was successfully exploited against West African human sleeping sickness (*Trypanosoma brucei gambiense*) (Bacchi et al., 1980; Burri and Brun, 2003). Interestingly, the functions of AdoMetDC and ODC are combined into a single unique bi-functional protein (*Pf* AdoMet/ODC) in *P. falciparum* (Müller et al., 2000). This enzyme has formed the basis of multiple studies assessing polyamine metabolism as a chemotherapeutic target in this organism. Inhibitors of this enzyme, however, have only cytostatic effects *in vitro* with cure achieved only with co-administration with polyamine analogs in murine malaria models (Bitonti et al., 1989).

Other polyamine analogs interfering with polyamine functions and metabolism have been synthesized and tested in different organisms. The tetraamines, homologs of spermine in which the external aminopropyl groups present in spermine were replaced by aminobutyl groups, are shown along with oligoamines to be effective in the treatment of microsporidiosis, an opportunistic infection associated with severe HIV infection (Bacchi et al., 2002). Spermine analogs were found to condense DNA and are indeed powerful inhibitors of human cell proliferation (Osland and Kleppe, 1977).

Based on these findings, we tested the oligoamines (SL-11158 and SL-11144) and the tetraamine SL-11093, with demonstrated efficacy against microsporidia, for their activities against malaria parasites. Here we report the identification of four lead compounds from the thiourea sub-series and one compound from the urea-based analogs from the list of twenty-two polyamine analogs effective against chloroquine-sensitive and -resistant parasites, at nanomolar levels. Moreover, these compounds were tested *in vivo* in a murine model with *Plasmodium yoelii* and found to significantly reduce levels of parasitemia. These effects are correlated with an observed decrease in spermidine and spermine levels and highlight the importance of this polyamine to the parasite.

## Methods

### Strains

The *P. falciparum* strains 3D7 and Dd2 used in this study were obtained from the Malaria Research and Reference Reagent Resource Center (MR4). *P. yoelii* clones were obtained from the WHO Registry Standard Malaria Parasites, University of Edinburgh.

### Chemicals

Hypoxanthine radiolabelled chemicals were purchased from NEN Life Science Products. Compounds SL-11144, SL-11158, SL-11091, and SL-11093 were synthesized by the Frydman group as described elsewhere (Reddy et al., 1998; Valasinas et al., 2003). All of the remaining compounds were synthesized by Dr. Patrick M. Woster, Medical University of South Carolina, as previously described (Zou et al., 2001; Bi et al., 2006; Verlinden et al., 2011; Verlinden et al., 2015).

### Cell Culture and Materials

*P. falcipar*um was cultured *in vitro* with erythrocytes using a gas mixture of 3% O_2_, 3% CO_2_, and 94% N_2_ as described by the method of Trager and Jensen (1976). Briefly, we used RPMI medium 1,640 supplemented with 30 mg/liter hypoxanthine (Sigma), 25 mM Hepes (Sigma), 0.225% NaHCO_3_ (Sigma), 0.5% Albumax I (Life Technologies, Grand Island, NY) and 10 μg/ml of gentamycin (Life Technologies) for the parasite growth. Synchronization of the parasites was obtained by incubation with 5% sorbitol treatments (Lambros and Vanderberg, 1979). The stage of the parasite was confirmed by fixed smears of the infected erythrocytes using Giemsa staining and observation by bright-field microscopy.

### Hypoxanthine Incorporation Assay

The susceptibility of parasites to different compounds was assessed by tritiated hypoxanthine uptake as described by Desjardins et al. (1979). Briefly, infected erythrocytes with 3% at the ring stages were incubated with the compounds from the list of twenty-two polyamine analogs at 0, 0.01, 0.05, 0.1, 1, 5, 10, 50, 100, 250, 500, and 750 μM in a medium free of hypoxanthine for 48 h. Two hundred microliter of the mixture was then added to a 96-well plate with 3*H*-hypoxanthine at a concentration of 0.5 μCi/well. The cells were incubated for 24 h, washed on an ultrafilter and radioactivity was counted using a scintillation counter. IC_50_ values are calculated from the sigmoidal inhibition curves using Prism and are represented in nM. Values are means of three independent experiments each performed in triplicate.

### Infection of Mice With Blood-Stage Parasites and Drug Treatments

Naive 8-weeks-old female Swiss Webster mice were intravenously infected with 2 × 10^5^ infected red blood cells (iRBCs) of *P. yoelii* YM parasites; 3 mice were included in each infection group. Two groups per treatment were performed. Drugs were dissolved in dimethyl sulfoxide (DMSO). For example, to inject 35 mg/kg, we first dissolved 35 mg of the drug in 1 mL of DMSO and then diluted it in 0.05% Tween 80 H_2_O, for a total of 10 mL. We then injected 200 μL of this solution into mice with a body weight of 20 g. The polyamine compounds and pyrimethamine were administered intraperitoneally. All mice were treated for 4 days. Parasitemia was monitored by Giemsa staining of blood smears obtained after the 4 days of treatment and on a daily basis. Parasites were harvested by collecting blood samples from the tail vein of infected mice. The mice tolerated (without observable toxicity) up to 35 mg/kg when the schedule described above was used. All mice studies were performed with the Institutional Animal Care and Use Committee at the Albert Einstein College of Medicine approval and oversight.

### Polyamine Quantification

Polyamine content was quantified as described previously (Bacchi et al., 2004). Briefly, *P. falciparum* Dd2 strain infected red blood cells were treated in the late schizont stage (42 h post invasion) with 1 μM of RHW (IC_50_ = 200 nM), 750 nM of compound 13 (IC_50_ = 150 nM), and with 5 mM DFMO (IC_50_ = 1 mM), to ensure complete parasite arrest. Treated and untreated cultures, at 10% parasitemia, were harvested at trophozoite phase (18–24 h). Polyamine interconversion was assayed by incubation infected red blood cells to 0.25 μCi (2.1 nmol) [1-^14^C]spermine as described previously by Bacchi et al. (2001). After incubation, mixtures were centrifuged and the supernatant was discarded. Pellets were extracted with 10% TCA overnight and frozen. Separation of polyamines was performed by HPLC equipped with a 5 μm C-18 reversed-phase column, samples and standards were detected by a UV detector (Perkin Elmer), and signals were integrated using a β-ram (IN/US Systems) Version 3.1 software package.

### Molecular Modeling

The graphical user interface Maestro (version 10.3, Schrodinger LLC, New York, NY, 2018) was used to visualize the *Pf* SpdS protein (PDB IDs: 2I7C and 4CWA), which was prepared using the Protein Preparation Wizard. Only chain A was used in both crystal structures for further modeling. The ligand RHW was prepared using the Maestro user interface. MacroModel force-field based molecular modeling (MacroModel v10.3, Schrodinger LLC, New York, NY, 2018) was used to predict the binding pose of the ligand RHW (Harder et al., 2015). The obtained molecular conformations were visualized using PyMoL (The PyMOL Molecular Graphics System, Version 1.8 Schrödinger, LLC).

## Results

### Effects of Polyamine Analogs on Growth of 3D7 and Dd2 of *P. falciparum* Strains

**Figure 1:**
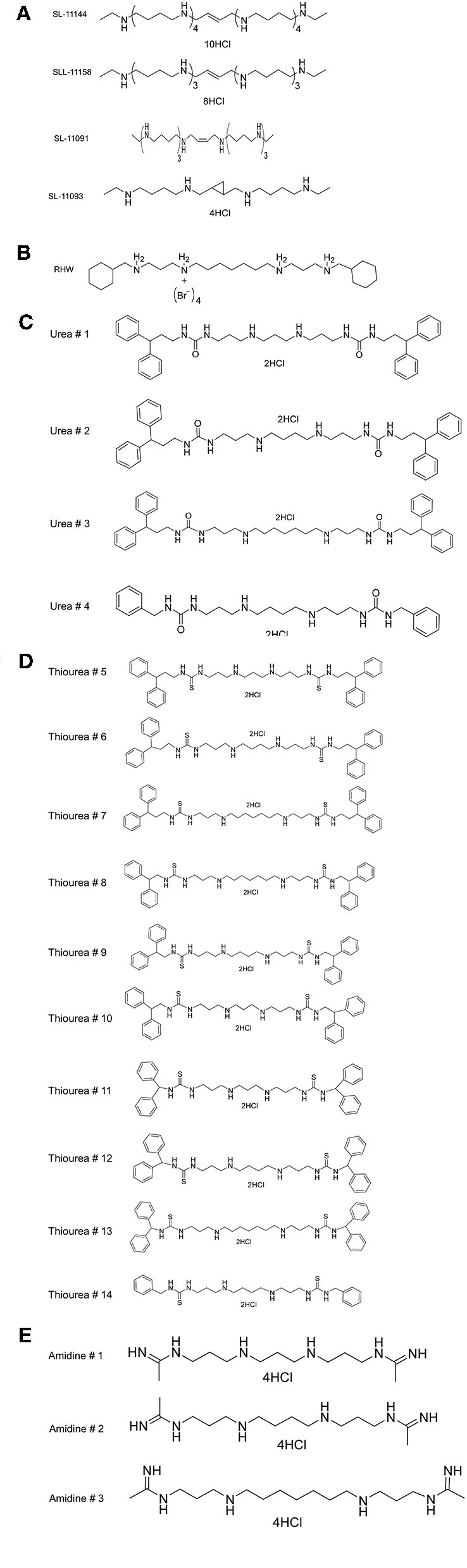
Structures of **(A)** various oligoamine and tetraamine analogs of polyamines, **(B)** *N*1, *N*7-bis (3-(cyclohexylmethylamino) propyl) heptane-1,7-diamine tetrabromide (RHW), **(C)** Urea, **(D)** Thiourea, **(E)** Amidine.

**Figure 2:**
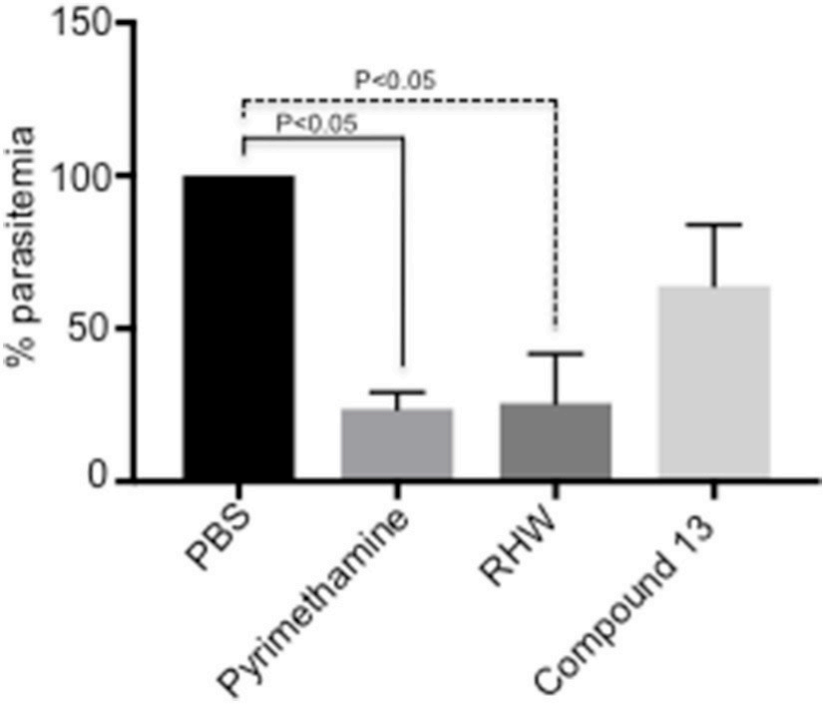
*In vivo* study of the effect on P. *yoelii*-infected mice (*n* = 3 animals per group) of polyamine analogs administered ip at 35 mg/kg/day as indicated in Methods. Pyrimethamine was administered ip as a positive control at 10 mg/kg/day. A control untreated group received PBS. Activity was determined by percentage of parasitemia at day 6-post infection relative to untreated control. The results are shown as the means of three independent experiments ± standard deviation. Significant differences relative to untreated control are determined by student's *t*-test.

Previous studies have shown that polyamine analogs with a backbone of repeating *N*-butyl subunits, such as pentamines, oligoamines or bis-(aryl)-substituted 3-7-3 analogs (Figure 1A), sterilized *Encephalitozoon cuniculi*-infected monolayer cells and cured two murine model infections (Bacchi et al., 2002). The IC_50_ of SL-11158 and SL-11144 against microsporidia was 8.2 and 0.62 μM (Bacchi et al., 2002). In order to examine the antimalarial activity of polyamine analogs, we have tested the effect of increasing concentrations of these compounds on the intraerythrocytic life cycle of *P. falciparum* in culture, by following the incorporation of radiolabeled hypoxanthine into parasite nucleic acids. We then determined the 50% inhibitory concentration (IC_50_) that blocks the replication of *P. falciparum* inside the red cells. The study was performed with two strains of *P. falciparum* (3D7 and Dd2) with different levels of sensitivity to the antimalarial drugs pyrimethamine and chloroquine (Figure 2 and Table 1). As a control, we confirmed the IC_50_ of chloroquine in the two strains. As expected, the strain 3D7 was sensitive to chloroquine with IC_50_ of 5 ± 0.2 nM and Dd2 was highly resistant to chloroquine (IC_50_ 300 ± 21 nM) (Supplementary Figure 1).

**Table 1:** Structure of various analogs of polyamines and their affect on the growth of 3D7 and Dd2 strains of *P. falciparum* under *in vitro* conditions.

**Compounds**	**No**.	**IC**_****50****_ (nM)^*^	
		**3D7 strains**	**Dd2 strains**
SL-11144		>250,000	>250,000
SL-11158		>250,000	>250,000
SL-11091		>250,000	>250,000
SL-11093		>250,000	>250,000
RHW		2,600 ± 135	200 ± 12
Urea	1	1,700 ± 95	800 ± 23
Urea	2	370 ± 4.8	440 ± 3.2
Urea	3	530 ± 7.2	630 ± 11
Urea	4	2,900 ± 37	700 ± 21
Thiourea	5	3,200 ± 19	3,600 ± 7.9
Thiourea	6	590 ± 53	510 ± 4.8
Thiourea	7	220 ± 2	350 ± 6.1
Thiourea	8	170 ± 10.5	160 ± 8
Thiourea	9	560 ± 2.3	690 ± 1.9
Thiourea	10	570 ± 6.0	850 ± 10.5
Thiourea	11	650 ± 29	1,950 ± 18
Thiourea	12	240 ± 6.8	460 ± 3.7
Thiourea	13	160 ± 10.4	150 ± 24
Thiourea	14	1,200 ± 8	1,720 ± 125
Amidine	15	>100,000	>100,000
Amidine	16	>100,000	>100,000
Amidine	17	>100,000	>100,000

* *IC_50_: The half maximal inhibitory concentration. IC_50_ values are calculated from the sigmoidal inhibition curves using Prism. IC_50_ values are an average of three independent experiments, each carried out in triplicate with ± standard deviations*.

As shown in Table 1 and Supplementary Figure 1, RHW exhibited antimalarial activity, with an IC_50_ of 2.6 μM against 3D7 and 0.2 μM against Dd2 parasite strains. This prompted the testing of a variety of polyamine analogs for antiparasitic effects. We tested 17 compounds belonging to three different classes, namely ureas, thioureas, and amidines (Figures 1C–E). The polyamines of the thiourea group were found to be the most potent in inhibiting parasite growth, with IC_50_ ranging from 160 to 3,200 nM for 3D7 strain and 150 to 3,600 nM for Dd2 strain (Table 1). Compound 13 was found to be more effective in inhibiting parasitic growth of both 3D7 and Dd2 strains with IC_50_ values of 160 nM and 150 nM, respectively. Both 3D7 and Dd2 parasite strains exhibited varied sensitivity toward the urea class of polyamine analogs, with compound 2 exhibiting the strongest growth inhibition of the class, with an IC_50_ of 370 nM for 3D7 and 440 nM for Dd2 strains (Table 1). The amidine class of analogs showed the least effect on growth of both strains of *P. falciparum* with an IC_50_ > 100 μM (Table 1). To further confirm the inhibitory effects of these classes of polyamine analogs on *P. falciparum* growth, we employed the pLDH colorimetric assay (data not shown), which measures parasite-specific lactate dehydrogenase activity (Makler and Hinrichs, 1993; Makler et al., 1993).

### Efficacies of Polyamine Analogs *in vivo*

We tested the efficacy of the most potent compounds on the growth inhibitory potential of *Plasmodium* employing a murine model. We infected mice with *P. yoelii*, as described in materials and methods, and the infected mice were treated with RHW and compound 13, the compounds with the most efficacy *in vitro*. PBS infected mice served as control and pyrimethamine drug treated animals were used as a positive control group. As shown in Figure 2, treatment of animals with RHW resulted in a significant (*p* < 0.05) decrease in parasitemia compared to the untreated control. Even though compound 13 decreased parasitemia *in vivo*, the level of inhibition was not significantly different (*p* > 0.05) to the PBS control group (Figure 2 and Supplementary Figure 2). In conclusion, these *in vivo* results show the efficacy of RHW on the inhibition of parasitemia in the murine model.

### Polyamine Quantification

**Figure 3:**
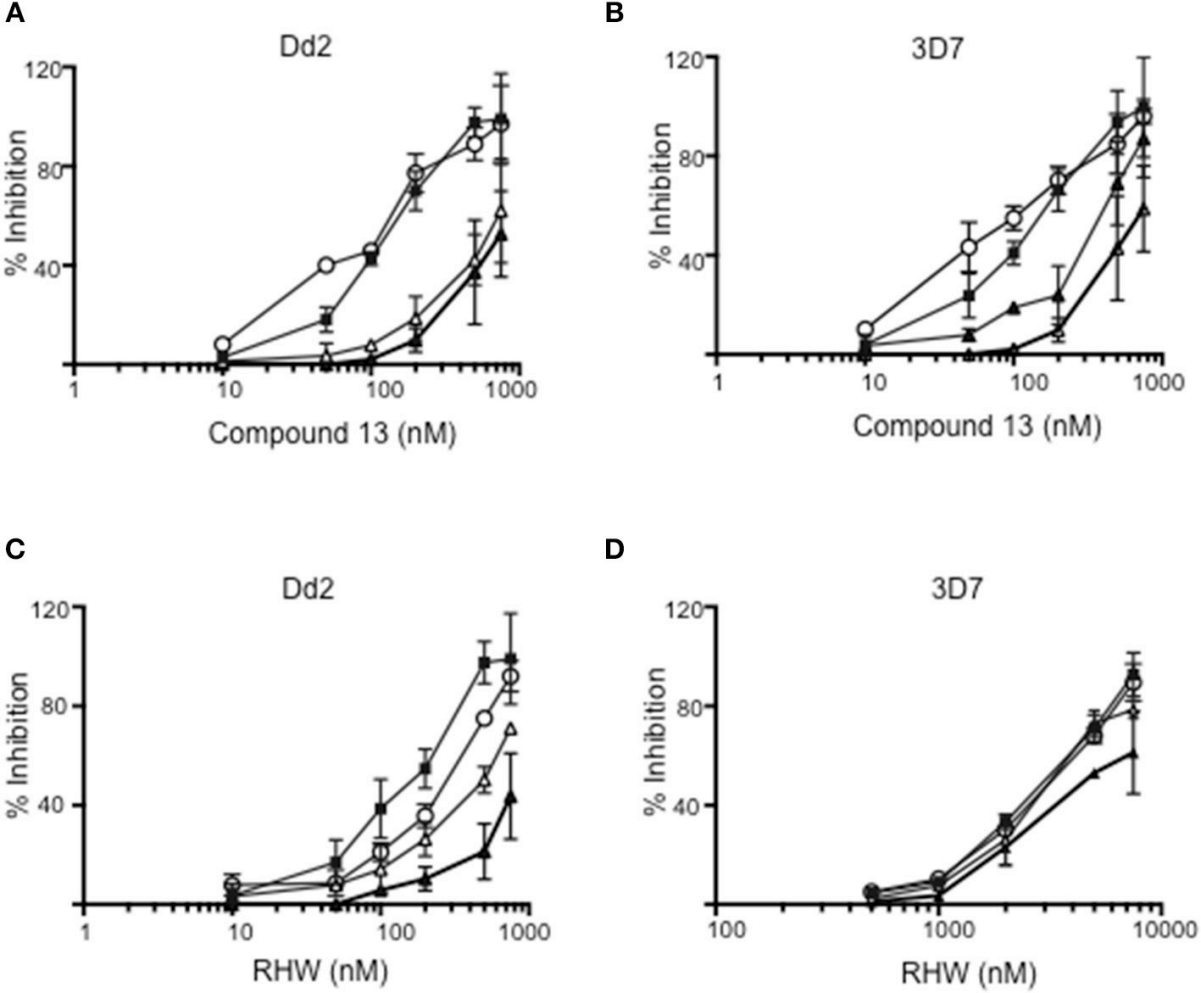
Inhibition of Dd2 and 3D7 strains by different concentrations of compound 13 **(A,B)** and RHW **(C,D)** in the absence of DFMO (closed squares), or in presence of 500 mM (open triangles), 1 mM (open circles), and 3 mM (closed triangles) of DFMO.

We examined the effects of polyamine analogs on overall uptake of arginine and putrescine, and production of spermidine and spermine. Table 2 shows the results of polyamine contents in red blood cells [experiment was performed twice, each in five replicate experiments, where the polyamine content of *Plasmodium* infected red blood cells was compared to treated cells with RHW, compound 13, and ornithine decarboxylase inhibitor difluoromethylornithine (DFMO) as a control]. We monitored the intracellular content of arginine, putrescine, spermidine, and spermine. Data show that DFMO substantially decreases the amount of spermidine. However, spermine levels were similar for both untreated and DFMO treated. These results were in concordance with previous data that show the same effect of DFMO (Sugiura et al., 1984). In contrast, infected red blood cells treated with RHW showed a significant decrease in spermidine (*p* < 0.05) and spermine levels (*p* < 0.01). These data suggest an effect of RHW on polyamine metabolism in *Plasmodium*, including an effect on enzymes involved on spermidine and spermine synthesis. In addition, when RHW or compound 13 was assayed in combination with DFMO in both Dd2 and 3D7 strains, the two drugs were found not to have synergistic antimalarial activity (Figure 3).

**Table 2:** Effect of treatment of RHW and compound 13 on the polyamine contents of *Plasmodium falciparum*.

**Sample**	**Amount (nmoles/ml)**			
	**Arginine**	**Putrescine**	**Spermidine**	**Spermine**
Infected untreated RBC	11.5 ± 3.9	8.0 ± 1.1	52.5 ± 1.9	13.0 ± 3.1
DFMO	32.5 ± 11	11.0 ± 2.4	32.0 ± 0.9	15.0 ± 2.9
Compound 13	12.0 ± 1.2	5.9 ± 1.7	50 ± 2.8	14.5 ± 1.7
RHW	41.0 ± 12.9	6.0 ± 2.1	41 ± 1.3	2.0 ± 0.8

*Plasmodium falciparum Dd2 strain infected red blood cells were treated in the late schizont stage with 1 μM of RHW (IC_50_ = 200 nM), 750 nM of compound 13 (IC_50_ = 150 nM), and with 5 mM DFMO (IC_50_ = 1 mM, as a positive control). The treated parasites were then extracted and monitored for intracellular polyamine levels. The results are presented as mean ± SD for 2 separate experiments, each performed in 5 replicates. Significant differences relative to untreated control are determined by student's t-test*.

### Molecular Modeling of Compound RHW

**Figure 4:**
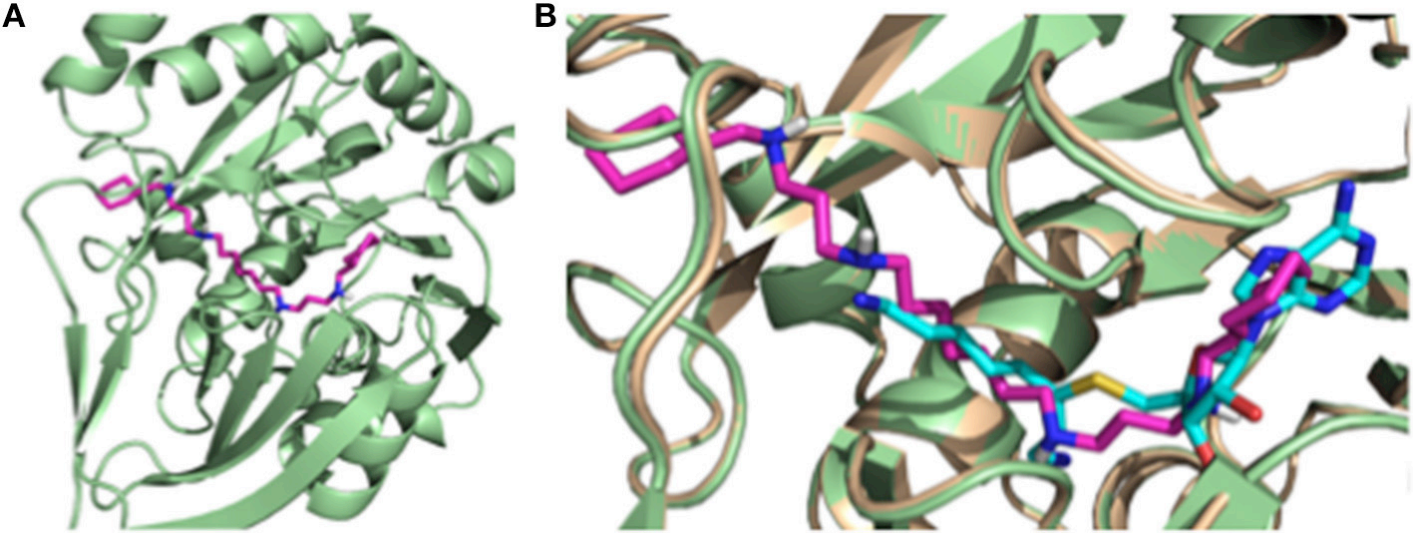
**(A)** Putative binding conformation of RHW (pink) in PfSpdS (green) traversing the three areas of the active site. **(B)** Overlay of RHW modeled conformation (pink) with 217C (wheat) and it's co-crystal inhibitor AdoDATO (sky blue).

In order to understand the putative binding conformations of RHW to *P. falciparum* spermidine synthase (*Pf* SpdS) and the observed biological activity, we utilized *in silico* molecular modeling. Sprenger et al. proposed three areas of the active site: the distal aminopropyl cavity, the putrescine site and the larger dcAdoMet site (composed of the central aminopropyl cavity and the MTA cavity) within *Pf* SpdS (Sprenger et al., 2016). Because RHW showed potent activity *in vitro* and *in vivo*, all our molecular modeling was performed with this inhibitor. RHW is a long linear molecule consisting of 20 rotatable bonds (Figure 1B). Attempts to dock and predict the binding mode of RHW using Schrodinger Glide XP mode were unsuccessful. Instead, we modified an existing ligand (5-(1H-benzimidazol-2-yl) pentan-1-amine) within the *Pf* SpdS active site of PBD ID: 4CWA (Sprenger et al., 2015), and subjected it to MacroModel minimization using force-field OPLS_2005, keeping the protein backbone rigid (locked) and allowing the side chains to move and generate a lower energy ligand:protein complex (Figure 4). RHW is predicted to bind across all three areas of the active site (Figure 4A), and when overlaid with inhibitor AdoDATO from crystal structure 2I7C (Dufe et al., 2007), RHW showed a similar mode of binding (Figure 4B). Some movement of the amino acid side chains (W234, C266, I235, H236, E231, M50, W51, and D178) was observed to accommodate the terminal cyclohexylmethyl groups. Of these, only H236 and D178 displayed significant movement (2.0 Å) of their side chain positions.

## Discussion

Multiple polyamine analogs in this study have demonstrated potent antimalarial properties, with IC_50_ at the nanomolar range for *P. falciparum* strains, both sensitive and resistant to chloroquine. Drug-resistance continues to present challenges in treating *P. falciparum* infection, and medications previously useful for treating drug-resistant isolates are increasingly inadequate, as illustrated by the emergence of artemisinin-resistance.

The utilization of polyamine analogs to treat infection has been previously demonstrated in experimental murine models of microsporidiosis, with several of the analogs demonstrating significant efficacy (Bacchi et al., 2004). The compounds with the most efficacies were shown to be tetraamines and oligoamines. These tetraamine compounds, which are structural homologs of spermine, have been studied extensively, and have been shown to interrupt and modify DNA conformational structure, inducing bends and kinks in the double helix (Hsieh et al., 1994). The oligoamine compounds have been demonstrated previously to contribute to the collapse of DNA, promoting condensation of DNA. These compounds have also documented anti-tumor activity (Frydman et al., 2003; Huang et al., 2003).

Given the importance of polyamine metabolism for transcriptional activity and cellular replication microsporidia and *Plasmodium*, testing analogs effective against microsporidia formed the basis of our initial approach toward *P. falciparum*. Surprisingly, these compounds showed no or minimal antimalarial activity. The lack of effect of the tetraamine compounds on *P. falciparum* prompted further synthetic efforts.

The addition of cyclohexylmethyl side groups to the tetraamine backbone resulted in the compound abbreviated as RHW. This compound showed substantial promise, with efficacy at the nanomolar range against chloroquine-resistant *P. falciparum*, and further medicinal chemistry allowed for additional modifications to these compounds. Some of these modifications proved beneficial, enhancing antimalarial activity, such as the urea and thiourea examples, while others were detrimental (amidine analogs). It has also been noted previously (Verlinden et al., 2011) that the compounds with aromatic rings demonstrated enhanced efficacy when compared to matched-pair compounds without these groups. The length of the carbon chain between the two central amines appears to also be important (see compounds 1–3). While the exact, mechanistic underpinning of this difference requires further exploration, this observation should inform any medicinal chemistry efforts with these polyamine analogs. We present a putative model of RHW bound to *P. falciparum* spermidine synthase (*Pf* SpdS) (Figure 4).

The observed differences in responsiveness to these compounds between microsporidia and *P. falciparum* highlight differences in the metabolic handling of polyamines by these two pathogens. It has been noted previously that microsporidia, including *E. cuniculi*, have an operative polyamine synthesis and interconversion pathway, the latter of which is lacking in *Plasmodium* (Müller et al., 2001). This difference in capacity could, in part, explain the differences observed in response to the tetraamine compounds. An alternative explanation could be structural differences in particular enzymes targeted by these compounds. The data presented here demonstrate decreased levels of spermine in infected cells and argue in favor of the polyamine analogs inhibiting spermidine synthase. While functional studies demonstrating this relationship are still needed, this seems a probable hypothesis for the mechanism by which the action of these compounds is exerted. Another potential target to inhibit polyamine synthesis is through blocking the transport of these molecules. Polyamine transporters have not yet been identified in the *P. falciparum* genome, but a drug combination selectively inhibiting both polyamine biosynthesis and transport may provide a promising anti-malarial strategy (van Brummelen et al., 2009).

The polyamine analogs investigated in this study have been demonstrated to hold promise as antimalarial agents and should be further characterized with respect to kinetics and impact on spermidine synthase. Moreover, following optimization, future studies with respect to efficacy, toxicity, and delivery to infected patients will be of value. Malaria is an enormous threat to the health of populations globally, responsible for millions of deaths annually. Novel therapeutic strategies are desperately needed in the continued fight against *Plasmodium*, and these polyamine analogs have the potential to represent one of these strategies, with the goal of more effectively treating infections and preventing the severe morbidity and mortality caused by this parasite worldwide.

## Author Contributions

KE, LW, and KK designed research. KE, HR, L-MT, RU, and NY performed research. KE, HR, L-MT, RU, NY, PW, JL, MM, LW, and KK analyzed data. KE, JL, KK, and LW wrote the paper. All authors read and approved the final manuscript version.

### Conflict of Interest Statement

The authors declare that the research was conducted in the absence of any commercial or financial relationships that could be construed as a potential conflict of interest.
