# Supplementary figures and images for: Novel Synthetic Polyamines Have Potent Antimalarial Activities in vitro and in vivo by Decreasing Intracellular Spermidine and Spermine Concentrations
Source: Front Cell Infect Microbiol. 2019 Feb 14;9:9. doi: 10.3389/fcimb.2019.00009 (PMC6382690; doi:10.3389/fcimb.2019.00009)

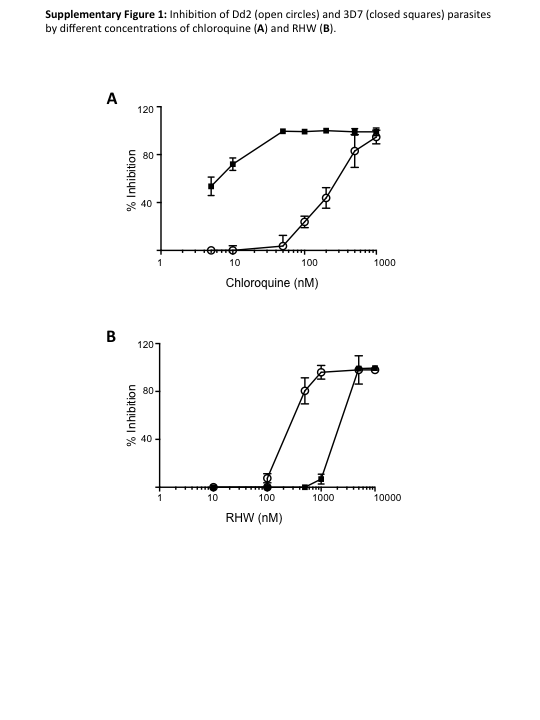

Supplement: Supplementary file 1 [file Image_1.TIF]

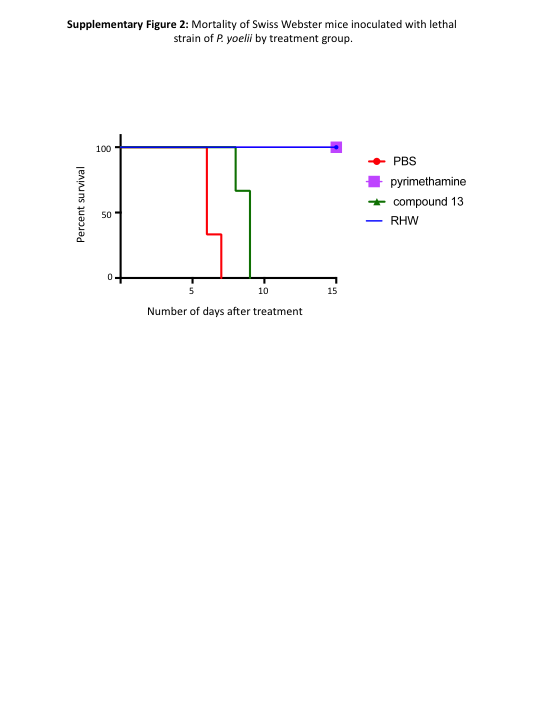

Supplement: Supplementary file 2 [file Image_2.TIF]
